# Biodegradation of DDT by *Stenotrophomonas* sp. DDT-1: Characterization and genome functional analysis

**DOI:** 10.1038/srep21332

**Published:** 2016-02-18

**Authors:** Xiong Pan, Dunli Lin, Yuan Zheng, Qian Zhang, Yuanming Yin, Lin Cai, Hua Fang, Yunlong Yu

**Affiliations:** 1 Institute of Pesticide and Environmental Toxicology, College of Agriculture & Biotechnology, Zhejiang University, Hangzhou 310029, China; 2 Division of Life Science, Hong Kong University of Science and Technology, Clear Water Bay, Hong Kong, China

## Abstract

A novel bacterium capable of utilizing 1,1,1-trichloro-2,2-bis(p-chlorophenyl)ethane (DDT) as the sole carbon and energy source was isolated from a contaminated soil which was identified as *Stenotrophomonas* sp. DDT-1 based on morphological characteristics, BIOLOG GN2 microplate profile, and 16S rDNA phylogeny. Genome sequencing and functional annotation of the isolate DDT-1 showed a 4,514,569 bp genome size, 66.92% GC content, 4,033 protein-coding genes, and 76 RNA genes including 8 rRNA genes. Totally, 2,807 protein-coding genes were assigned to Clusters of Orthologous Groups (COGs), and 1,601 protein-coding genes were mapped to Kyoto Encyclopedia of Genes and Genomes (KEGG) pathway. The degradation half-lives of DDT increased with substrate concentration from 0.1 to 10.0 mg/l, whereas decreased with temperature from 15 °C to 35 °C. Neutral condition was the most favorable for DDT biodegradation. Based on genome annotation of DDT degradation genes and the metabolites detected by GC-MS, a mineralization pathway was proposed for DDT biodegradation in which it was orderly converted into DDE/DDD, DDMU, DDOH, and DDA via dechlorination, hydroxylation, and carboxylation, and ultimately mineralized to carbon dioxide. The results indicate that the isolate DDT-1 is a promising bacterial resource for the removal or detoxification of DDT residues in the environment.

1,1,1-trichloro-2,2-bis(p-chlorophenyl)ethane (DDT) is an organochlorine pesticide that has been used extensively since the 1940s for the control of agricultural pests and vector-borne diseases like malaria and typhus^1^. DDT is toxic and recalcitrant to degradation with the half-life of 4–30 years. More seriously, its major metabolites 1,1-dichloro-2,2-bis(p-chlorophenyl)ethane (DDD) and 2,2-bis(p-chlorophenyl)-1,1-dichlorethylene (DDE) are more toxic and recalcitrant than the parent compound^2^. Although DDT was officially banned for use in China for over 30 years because of its high toxicity, long persistence, and high bioaccumulation, DDT isomers and their major metabolites can still be frequently detected in soils, sediments, surface water, and groundwater in recent years^3^,^4^. As priority persistent organic pollutants (POPs) and endocrine-disrupting chemicals (EDCs), the exposure to DDT can cause a wide range of acute and chronic effects including carcinogenesis, estrogenic action, and endocrine disruption, posing a serious risk to environmental and human health^5^,^6^,^7^. As a result, there is an increasing concern on environmental DDT contamination and an increasing interest in DDT remediation.

Biodegradation has been considered as a cost-effective, safe, and promising method for the removal or detoxification of DDT residues in the environment^8^,^9^. The degradation characteristics of DDT by microorganisms have been well documented, and some DDT-degrading microorganisms have been isolated, such as *Alcaligenes eutrophus* A5, *Alcaligenes* sp. KK, *Bacillus* sp. BHD-4, *Cladosporium* sp. AJR^3^ 18501, *Pseudomonas* sp. 12–3, *Serratia marcescens* DT-1P, *Stenotrophomonas* sp. 103–105, *Sphingobacterium* sp. D6, and white-rot fungi^10^,^11^,^12^. The biodegradation mechanisms of DDT have also been conducted in several studies to detect the generated metabolites using GC-MS. The strain *Sphingobacterium* sp. D-6 could convert DDT into six metabolites (DDE, DDMU, DDNS, DDA, DBP, and 3-hydroxyl-2-(*p*-chlorophenyl) propionic acid) and then mineralize to carbon dioxide in mineral salts medium (MSM)^13^. The members of *Pseudomonas aeruginosa* and *Flavimonas oryzihabitans* could transform DDT into DDE, DDMU and DDOH in broth medium^14^. The strain *Rhodococcus* sp. IITR03 could degrade DDT to DDE, DDD, and DDMU in minimal medium^1^. Presently, whole genome sequencing and functional annotation of the DDT-degrading isolate is a promising approach to find out functional genes and understand their roles in the degradation pathways. To the best of our knowledge, DDT biodegradation mechanisms have not been explored by integrated genomic and GC-MS analyses.

In the present study, a bacterial strain DDT-1 capable of utilizing DDT as the sole source of carbon and energy was isolated, purified, and identified. The objectives of this study were: 1) to measure physiological characterization of the isolate DDT-1; 2) to conduct genome sequencing and analysis of the isolate DDT-1; 3) to examine the effect of substrate concentration, pH, and temperature on the degradation of DDT by the isolate DDT-1; and 4) to reveal the potential DDT biodegradation pathway by DDT degradation genes database (DDG) search and GC-MS analysis. The results will provide a new insight into the understanding of DDT biodegradation mechanisms.

## Results and discussion

### General characterization of the strain DDT-1

**Figure 1:**
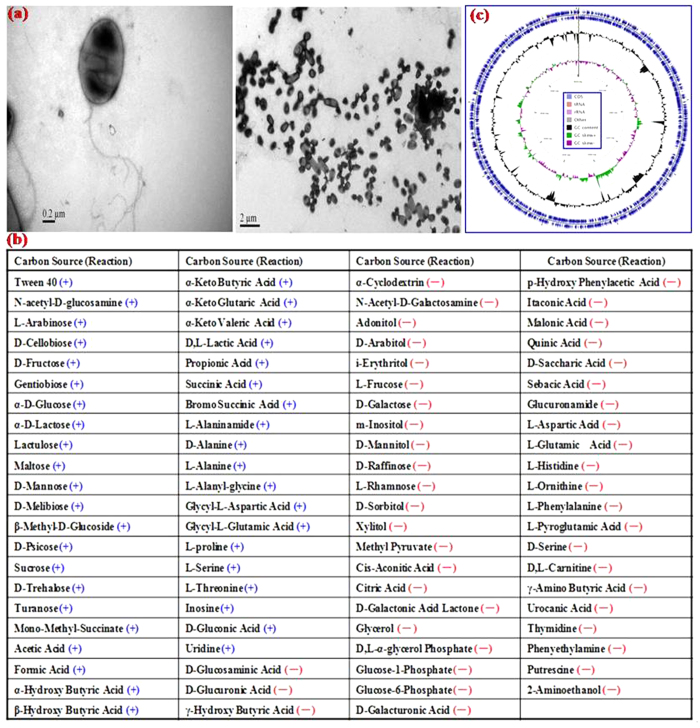
Scanning electron microscope (**a**), Metabolic profile (**b**), and graphical circular genome map (**c**) of the isolate DDT-1.

A bacterial strain, designated as DDT-1, with the capability to utilize DDT as the sole carbon and energy source was isolated from the DDT-contaminated soil. Growth curve of the isolate DDT-1 in MSM (pH 7.0) containing 1.0 mg/l of DDT is shown in Fig. S1. Scanning electron microscope (SEM) photos of the isolate DDT-1 is shown in Fig. 1a. The cells were ellipse-shaped aerobic bacterium with size of 0.5–0.7 μm × 0.9–1.1 μm. It was yellow, bacterial opacity, edge neat, smooth and moist, surface uplift on the LB plate, and could grow in MSM containing 1.0 mg/l of DDT at temperature 15–35 °C and at pH 5.0–9.0. The isolate DDT-1 were Gram-negative and tested positive for catalase, gelatin hydrolysis, and lysine decarboxylase, but negative for oxidase, nitrate reduction, and starch hydrolysis. The relative utilization capacities of 95 different substrates by the isolate DDT-1 in BIOLOG GN2 microplate are given in Fig. 1b. The isolate DDT-1 was found to be the most closely related to the genus *Stenotrophomonas* with 0.74 similarity (24 h) using BIOLOG Microlog 4 database software. As shown in Table S1, its 16S rDNA sequence had a high similarity (99%) to the members of the genus *Stenotrophomonas*. According to the above identifications, the strain was designated as *Stenotrophomonas* sp. DDT-1.

### Genome properties

Genome properties of the isolate DDT-1 are shown in Table 1 and Fig. 1c. The detailed genomic information annotated against COGs, GO, and KEGG pathway databases is summarized in Tables S2-S4. As shown in Table 1, a total of 1,496,317,952 bp clean data were filtered from 1,767,899,136 bp of raw data. These clean sequences were assembled into 97 contigs (>500 bp, Table S5) with a genome size of 4,514,569 bp, GC content of 66.92%, and more than 300 fold genome coverage. Totally, 4,108 genes were annotated, 4,033 of which were protein-coding genes. The remaining 75 genes were RNA genes including 8 rRNA genes. There were 2,807, 1,582, and 1,601 protein-coding genes that were assigned to COGs, catalytic categories, and KEGG pathways, respectively.

### Effect of substrate concentration on the biodegradation of DDT

**Figure 2:**
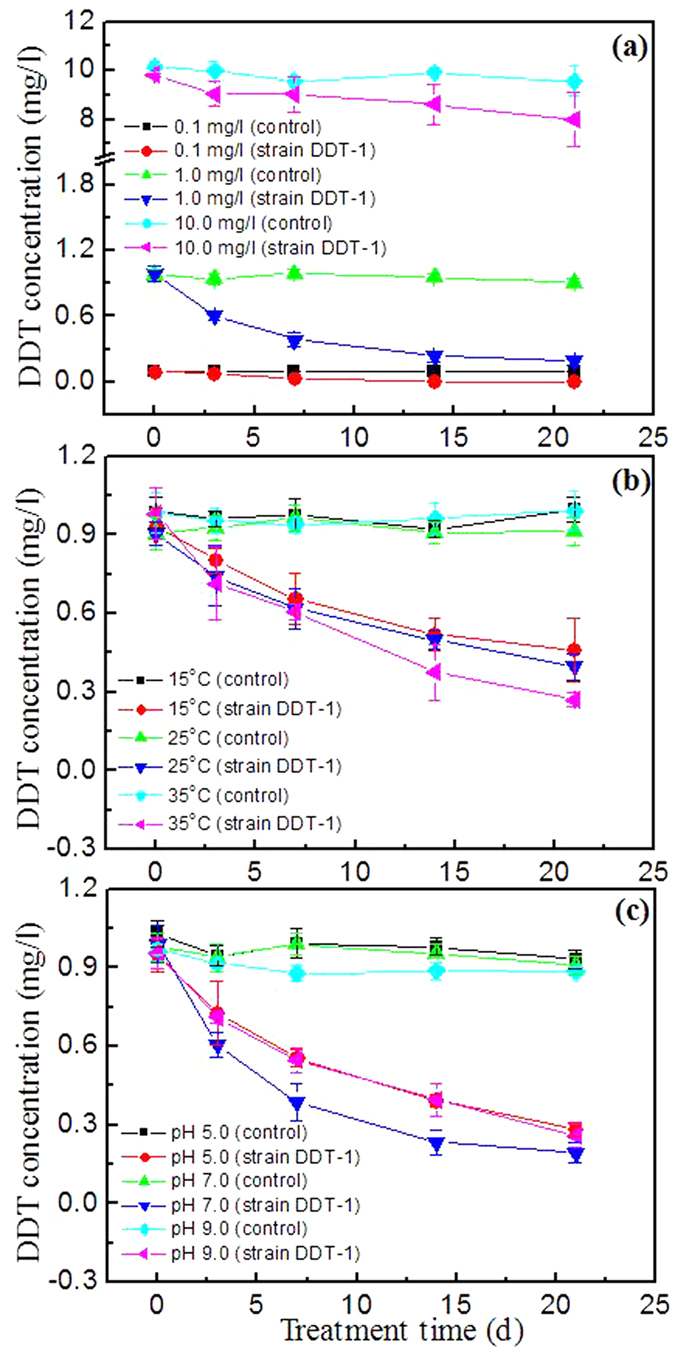
Effects of substrate concentration (**a**), temperature (**b**), and pH (**c**) on the degradation of DDT by the isolate DDT-1 in mineral salts medium. Error bars indicate the standard deviations with n = 3.

The degradation effects of different concentrations of DDT by the isolate DDT-1 in MSM of pH 7.0 at 25 °C is shown in Fig. 2a and kinetic data are summarized in Table 2. In all controls without the isolate DDT-1, the hydrolysis percentages of DDT were less than 7.5%. After incubation for 21 d, the degradation rate of DDT at three concentrations of 0.1, 1.0, and 10.0 mg/l was 0.004, 0.038, and 0.086 mg/l/d, respectively (Table 2). The degradation rate of DDT increased almost linearly with increasing concentrations (*r* = 0.94), suggesting that the degradation is subjected to pseudo first-order kinetics. As shown in Table 2, the degradation half-lives of DDT at concentrations of 0.1, 1.0, and 10.0 mg/l were 4.0, 8.1, and 82.5 d, respectively. The ANOVA analysis showed that the degradation half-lives of DDT obviously increased with increasing concentration from 0.1 to 10.0 mg/l. In agreement with our results, 82.63% of 100 mg/l of DDT in medium was degraded by the mixed culture of six isolates belonging to the genera *Bacillus*, *Staphylococcus*, and *Stenotrophomonas* for 31 days at 30 °C^15^. About 65% of DDT (50 mg/l) in the coffee bean culture medium was degraded by *Flavimonas oryzihabitans* and *Pseudomonas aeruginosa* at the biomass level of OD_600_ = 0.6 for 7 days at 30 °C^14^. The degradation of DDT (100 mg/l) by the isolate *Stenotrophomonas maltophilia* AJR^3^ 9,504 at an initial population size of 10^5^–10^6^ cells/ml in basal salts medium containing 1.0 g/l of peptone resulted in a 35% decrease in DDT concentration after 28 days^16^. DDT removal at 150 mg/l by immobilized *Pseudomonas fluorescens* in a glass column packed with tezontle was as high as 55–99%^17^.

### Effect of temperature on the biodegradation of DDT

The effect of various temperatures on the degradation of 1.0 mg/l of DDT by the isolate DDT-1 in MSM at pH 7.0 is shown in Fig. 2b, and kinetic data of DDT degradation are summarized in Table 2. In all controls without the isolate DDT-1, the hydrolysis percentages of DDT were less than 1.5%. As shown in Table 2, the degradation half-lives of DDT at the level of 1.0 mg/l were 17.7, 15.6, and 11.5 d at 15, 25, and 35 °C, respectively, and the corresponding degradation rate was 0.022, 0.024, and 0.034 mg/l/d. The ANOVA analysis showed that the degradation rate of DDT increased with temperature from 15 °C to 35 °C, and the highest degradation rate of DDT was observed at 35 °C. A result was reported by Gunasundari and Muthukumar^18^, who showed that the degradation rate of phenol by *Stenotrophomonas* sp. strain in M9 minimal medium increased with temperature from 28 °C to 35 °C.

### Effect of pH on the biodegradation of DDT

The degradation of DDT (1.0 mg/l) by the isolate DDT-1 in MSM of pH 5.0, 7.0, and 9.0 is shown in Fig. 2c. Throughout all experiments, less than 10% DDT was hydrolyzed in the controls without the isolate DDT-1 addition. As shown in Table 2, the degradation half-lives of DDT were 12.0, 7.6, and 11.1 d at pH 5.0, 7.0, and 9.0, respectively, and the corresponding degradation rate was 0.032, 0.038, and 0.033 mg/l/d. The ANOVA analysis showed that the degradation half-lives of DDT at pH 7.0 were statistically significantly (*p* ≤ 0.05) shorter than those at pH 5.0 and 9.0. The results indicated that neutral condition was favorable for the degradation of DDT by the isolate DDT-1. A similar result was also reported by Fang *et al.*^13^, who showed that neutral condition was the most suitable for the degradation of DDTs by the strain *Sphingobacterium* sp. D6 in MSM. However, Xie *et al.*^11^ found the highest degradation rate of DDT by *Alcaligenes* sp. KK at weak acidic condition (pH 6.0).

### Biodegradation pathway of DDT revealed by genome annotation

**Figure 3:**
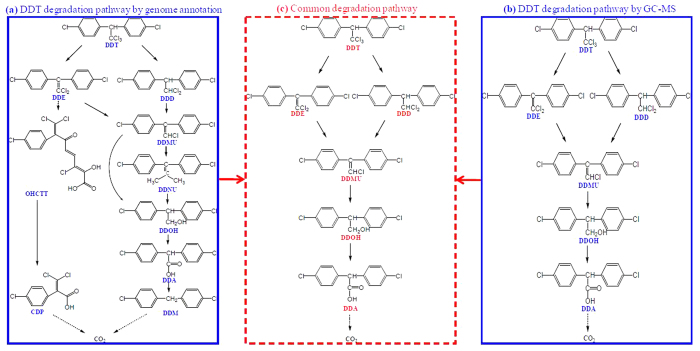
The potential degradation pathway of DDT by the isolate DDT-1 based on genome annotation (**a**). The proposed degradation pathway of DDT by the isolate DDT-1 based on GC-MS analysis (**b**). The common degradation pathway of DDT based on genome annotation and GC-MS analysis (**c**). DDT: 1,1,1-trichloro-2,2-bis(*p*-chlorophenyl)ethane; DDD: 1,1-dichloro-2,2-bis (*p*-chlorophenyl)ethane; DDE: 2,2-bis(*p*-chlorophenyl)-1,1-dichlorethylene; DDMU: 1-chloro-2,2-bis(4′-chlorophenyl)ethylene; DDNU: unsym-bis(4′-chlorophenyl)ethylene; DDOH: 2,2-bis(4′-chlorophenyl)ethanol; DDA: bis(4′-chlorophenyl)acetate; DDM: bis(4′-chlorophenyl)methane.

Potential DDGs that hit the isolate DDT-1 genome are summarized in Table S6. As shown in Table S6, these identified DDGs in the DDT-1 genome played roles in terms of DDT→DDE (*dhc* gene) by dehydrochlorination, DDT→DDD by dechlorination (*rdh* gene), DDMU→DDOH by hydrogenation and hydroxylation (*sds* and *dhg* genes), DDNU→DDOH by hydroxylation (*hdt* gene), DDA→DDM by decarboxylation (*dcl* gene), and OHCTT→CDP by carboxylation (*hdl* gene). As shown in Fig. 3a, the potential degradation pathways of DDT by the isolate DDT-1 were revealed by BLAST search against DDGs database. The isolate DDT-1 could convert DDT into DDE and DDD by dechlorination at the trichloromethyl group, and then into DDMU and DDNU by further dechlorination, and then into DDOH by hydroxylation, which further transform to DDA and DDM by carboxylation and decarboxylation, and ultimately mineralize to carbon dioxide. Simultaneously, the isolate DDT-1 could also convert DDT into OHCTT by dioxygenation and subsequent *meta*-ring cleavage, and then into CDP by carboxylation, and ultimately mineralize to carbon dioxide. Similar biodegradation pathways of DDT using metagenomic analysis was also revealed by Fang *et al.*^19^, who reported that the potential DDT biodegradation pathway in freshwater and marine sediments was a multistep process involving dechlorination, hydrogenation, dioxyenation, hydroxylation, decarboxylation, hydrolysis, and *meta*-ring cleavage reactions.

### Biodegradation pathway of DDT revealed by GC-MS analysis

**Figure 4:**
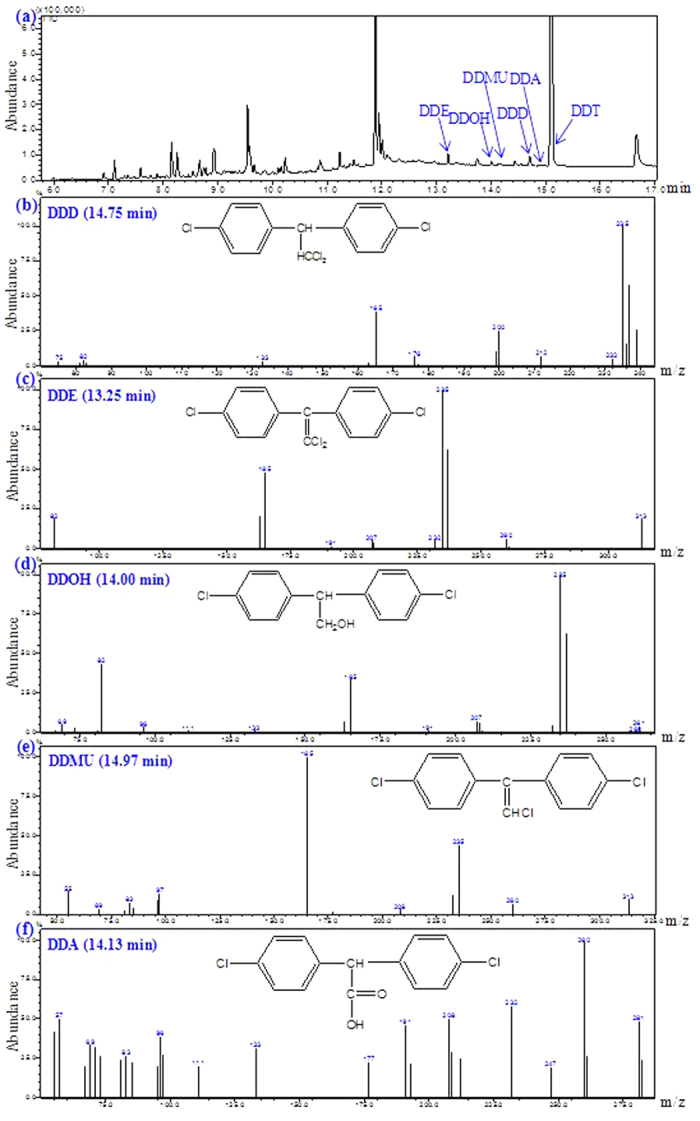
Total ion chromatogram (TIC) (**a**) and mass spectra (**b**–**f**) of DDT metabolites by the isolate DDT-1 in mineral salts medium.

Total ion chromatogram (TIC) and mass spectra of DDT metabolites by the isolate DDT-1 in MSM are shown in Fig. 4. Molecular weight and characteristic peak of DDT metabolites are summarized in Table S7. After inoculation for 14 d, five metabolites of DDT were identified with mass ion at *m/z* of 320, 318, 266, 284, 282 in MSM by GC-MS (Fig. 4a). Referring to known standard compounds and reported DDT metabolites, these five metabolites were identified as DDD at *m/z* 320 (C_14_H_10_Cl_4_, Fig. 4b), DDE at *m/z* 318 (C_14_H_8_Cl_4_, Fig. 4c), DDOH at m/z 266 (C_14_H_12_Cl_2_O, Fig. 4d), DDMU at *m/z* 284 (C_14_H_9_Cl_3_, Fig. 4e), and DDA at *m/z* 282 (C_14_H_10_Cl_2_O_2_, Fig. 4f), respectively. The compounds corresponding to the other peaks in TIC were not identified which should not be the metabolites of DDT. In addition, the accumulation of these metabolites was not observed by the determination in MSM during incubation for 14 d, suggesting that the isolate DDT-1 could mineralize DDT to carbon dioxide through these five metabolites as intermediates.

Based on these metabolites detected by GC-MS, a potential biodegradation pathway was proposed: DDT was initially converted into DDE and DDD by dechlorination at the trichloromethyl group, and then into DDMU, which was further transformed to DDOH and DDA by hydroxylation and carboxylation, and finally mineralized to carbon dioxide (Fig. 3b). Several similar studies on the biodegradation mechanism of DDT have been conducted by GC-MS. Rangachary *et al.*^20^ reported that the strain *Pseudomonas putida* T5 could transform DDT to DDE or DDD by eliminating either HCl or Cl^-^. Fang *et al.*^13^ reported that the strain *Sphingobacterium* sp. D-6 could dechlorinate DDT to DDD, DDE, DDMU, and DDNS, and then to DDA and DBP by hydroxylation, carboxylation, and decarboxylation, and finally mineralize to carbon dioxide. Xiao *et al.*^10^ reported that both *Phlebia lindtneri* GB-1027 and *Phlebia brevispora* TMIC34596 could metabolize DDT to DDD, DDA, and DBP. Additionally, a similar biodegradation pathway in coastal sediment was also reported by Yu *et al.*^21^, who showed that DDT was initially transformed to DDD and DDE, and then to DDMU and DDNU.

### DDT degradation pathway confirmed by both genomic and GC-MS analyses

**Figure 5:**
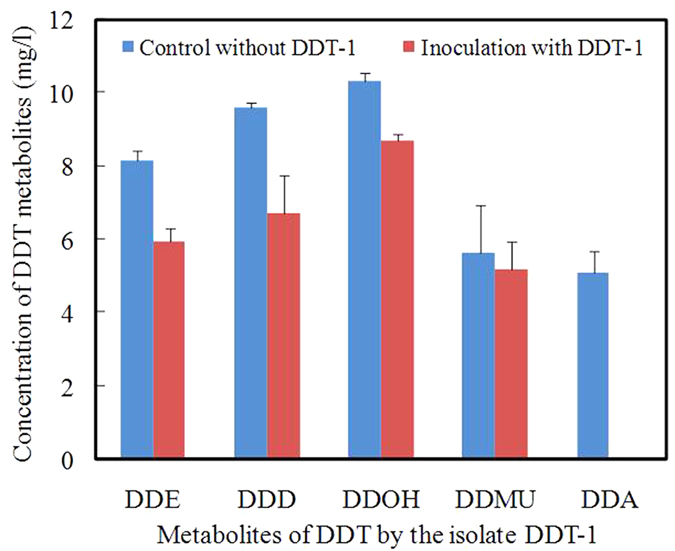
Degradation of DDT metabolites by the isolate DDT-1 in mineral salts medium after incubation for 7 d. Error bars indicate the standard deviations with n = 3.

The shared degradation pathway of DDT by the isolate DDT-1 between genome annotation and GC-MS analysis is shown in Fig. 3c. As shown in Fig. 3c, DDT was converted into DDE, DDD, and DDMU by dechlorination at the trichloromethyl group, and then to DDOH by hydroxylation, which was further transformed to DDA by carboxylation, and was ultimately mineralized to carbon dioxide. To confirm the degradability of DDT metabolites by the isolate DDT-1, biodegradation experiments (10.0 mg/l for each DDT metabolite) were conducted in MSM at pH 7.0, 150 rpm, and 30 °C. After incubation for 7 d, the degradation percentages of DDE, DDD, DDOH, DDMU, and DDA were 18.5%, 4.1%, 3.4%, 43.5%, and 49.2% in the controls without the isolate DDT-1 and 40.4%, 32.9%, 13.1%, 48.1%, and 100% in the inoculated treatments with the isolate DDT-1 (Fig. 5). The results showed the higher degradation rate in the inoculated treatments than in the un-inoculated controls, which indicated that these DDT metabolites could be degraded by the isolate DDT-1. In addition, the high degradation rate of DDMU and DDA in the un-inoculated controls indicated that these two metabolites were easily hydrolyzed or transformed in the MSM.

## Methods

### Chemicals

Standard samples of *p,p’*-DDT (purity 99.5%), *p,p’*-DDD (purity 98.0%), *p,p’*-DDE (purity 98.5%), DDMU (purity 99.0%), DDOH (purity 99.5%), and DDA (purity 99.0%) were purchased from Dr. Ehrenstorfer GmbH (Augsburg, Germany). Sodium chloride, anhydrous sodium sulfate, and *n*-hexane of analytical grade were provided by Sinopharm Chemical Reagent Co., Shanghai, China. *n*-Hexane of chromatographic grade was provided by Tedia Co., USA.

### Isolation and purification of the DDT-degrading microorganism

Soil samples were collected from the vegetable field located in Cixi, Zhejiang, China. These soil samples were homogenized and divided into three subsamples (10.0 g). Each subsample was mixed thoroughly with 100 ml of sterilized distilled water in 250-ml Erlenmeyer flask. Subsequently, an aliquot of 1.0 ml from soil suspension was inoculated into a 100-ml Erlenmeyer flask containing 20 ml of sterile mineral salts medium (MSM; MgSO_4_·7H_2_O, 0.40 g; FeSO_4_·7H_2_O, 0.002 g; K_2_HPO_4_, 0.20 g; (NH_4_)_2_SO_4_, 0.20 g; CaSO_4_, 0.08 g; H_2_O, 1000 ml; pH 7.0) supplemented with 1.0 mg/l of DDT as the sole source of carbon and energy. Each culture was incubated for 1 week on a rotary shaker at 30 °C and 150 rpm, and one loop of the culture was inoculated into 20 ml of MSM supplemented with 2.0 mg/l of DDT and incubated for another 1 week under the same conditions. The culture was repeatedly acclimated in fresh MSM with increasing concentration of DDT from 2.0 to 10.0 mg/l. Subsequently, one loop of the enrichment culture was streaked onto MSM agar plates containing 10.0 mg/l of DDT and incubated for 6 d at 30 °C, and individual pure colonies were then restreaked in fresh MSM agar plates and incubated for another 6 d. Once pure cultures were achieved, they were stored for further study.

### BIOLOG assay

Carbon substrate utilization for the isolate DDT-1 was assessed using BIOLOG GN system (BIOLOG Inc., Hayward, CA, USA). The isolate DDT-1 was incubated on BUG (BIOLOG Universal Growth Agar) at 30 °C for 24 h prior to assay. The fresh colonies were removed from BUG media with a long cotton swab and suspended in 15 ml of inoculation fluid with turbidity equivalent to 72% transmittance as measured by BIOLOG turbidimeter. Each well in the BIOLOG GN2 microplate^TM^ was inoculated with 150 μl of bacterial suspension using an 8-channel pipette, and then incubated in a biochemical incubator at 25 ± 1 °C. Color development in the wells was monitored at 6 h and 24 h, respectively, using a microplate reader with a 590 nm filter (BIO-TEK Instruments, Winooski, VT, USA). The output data were recorded by the automatic threshold option using BIOLOG software (BIOLOG Gen III database v2.7).

### DNA preparation, 16S rDNA and genome sequencing

DNA was extracted from 1 ml of suspension of the isolate DDT-1 using a QIAamp DNA mini kit (Qiagen, Hilden, Germany) according to the manufacturer’s protocol. The concentration and quality of the extracted DNA were examined with spectrophotometry (NanoDrop ND-1000, Wilmington, DE, USA). The 16S rDNA was amplified using PCR with an Eppendorf Thermocycler (Eppendorf, Hamburg, Germany) in a volume of 25 μl containing 1 μl of template DNA (20 ng/μl), 2.5 μl of 10×PCR buffer, 2 μl of MgCl_2_(15 mmol/l), 2 μl of dNTPs(2.5 mmol/l), 1 μl of primers (10 μmol/l, universal primer pair: 27F 5′-AGAGTTTGATCCTGGCTCAG-3′; 1492R 5′-GGTTACCTTGTTACGACTT-3′), 0.25 μl of Taq DNA polymerase (5 U/μl), and 16.25 μl of ultrapure water. Amplification conditions were as follows: an initial denaturation step for 5 min at 94 °C, 34 cycles of denaturation for 30 s at 94 °C, annealing for 45 s at 58 °C, extension for 90 s at 72 °C, followed by a final extension for 5 min at 72 °C. The PCR products were purified and then sequenced using an ABI 3730 automated DNA sequencer (Invitrogen Biotechnology, Shanghai, China). Sequences obtained were assembled and then compared with the NCBI GenBank sequences using online BLASTn search.

Prepared genomic DNA samples were sent to Novegene (Beijing, China), and approximately 5 μg of DNA samples was used for shotgun library construction. DNA was first mechanically fragmented with an enrichment size of ~500 bp. The DNA fragments were recycled by gel purification and quality check, and were then used for shotgun library construction, which was finally used for genome sequencing using an Illumina MiSeq platform with the paired-end 250 bp sequencing strategy. Approximately 1.77 Gb of raw reads were generated for the isolate DDT-1.

### Database construction

Protein database of DDT degradation genes (DDGs) for complete metabolic pathways containing 12 sub-databases (*dhc*, *rrat*, *sds*, *cpo*, *doa*, *dcl*, *ort*, *hdl*, *ods*, *rdh*, *dhg*, and *hdt*) was retrieved from the KEGG pathway maps (http://www.genome.jp/kegg/). DDGs database was deduplicated to obtain 982 non-redundant proteins (Table S8).

### Genome annotation

**Figure 6:**
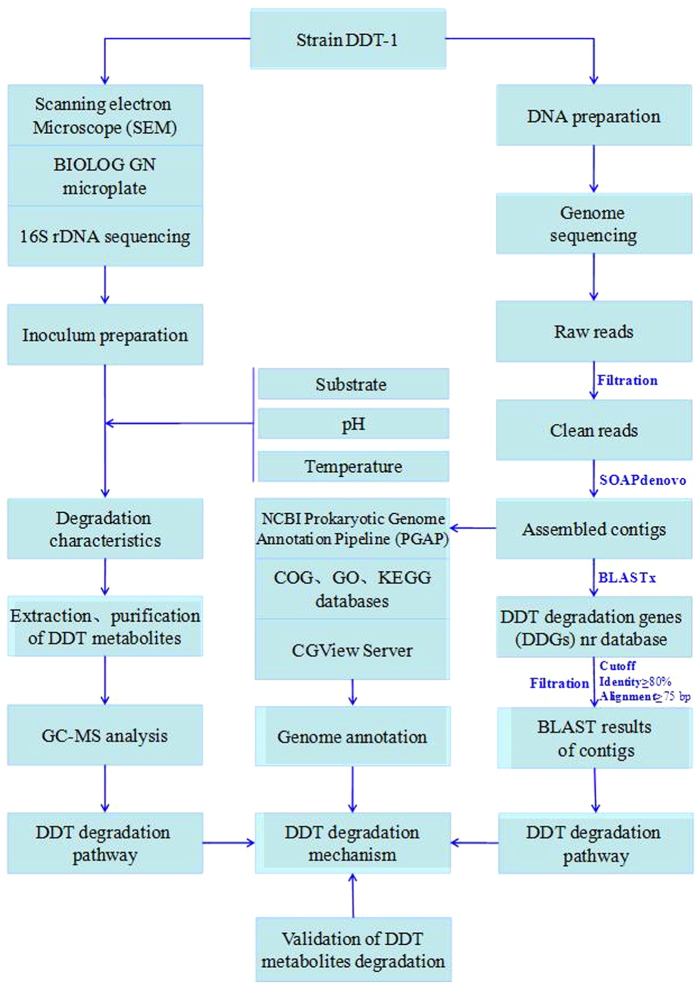
The flow chart of this study.

The flow chart of this study is shown in Fig. 6. The raw dataset was filtered using a self-written script to remove the reads containing three or more ambiguous nucleotides and those with quality value lower than 20. Then, the paired-end clean reads were assembled into contigs with SOAPdenovo using a Kmer of 29 bp. The obtained contigs were aligned against the established DDGs database using BLASTx with the E-value cut-off at 10^−6^, and then the best hit results were filtered with the cut-off at identity (≥80%) and alignment length (≥25 aa) by a self-written Python script.

The draft genome was annotated by GeneMarkS+ tool according to NCBI Prokaryotic Genome Annotation Pipeline (PGAP). The genomic functional annotation of the isolate DDT-1 was conducted by Reversed Position Specific BLAST (RPS BLAST) program search against the Clusters of Orthologous Groups (COGs) database with an E-value cutoff (1e^−10^)^22^. Kyoto Encyclopedia of Genes and Genomes (KEGG) mapping of the isolate DDT-1 was conducted against KEGG pathway database, and cellular component, molecular function, and biological process of the isolate DDT-1 were annotated by InterProScan search against Gene Ontology (GO) database^23^. All obtained contigs were joined together by CLC Main Workbench (version 7.6.2, CLC Bio, Aarhus, Denmark), which was then submitted to CGView Server to plot graphical circular map of the isolate DDT-1 (http://stothard.afns.ualberta.ca/cgview_server/)^24^.

### Degradation of DDT by the isolate DDT-1 in pure culture

The isolate DDT-1 was pre-cultured in 250-ml Erlenmeyer flasks containing 150 mL of LB medium at 30 °C and 150 rpm on a rotary shaker. The cells at the exponential phase were harvested by centrifugation (8,000×g, 10 min), and then immediately washed 3 times with 20 mL of NaH_2_PO_4_-Na_2_HPO_4_ buffer (0.1 mol/l, pH 7.0), and finally suspended in the same phosphate buffer as the seed. In order to study the degradation of DDT by the isolate DDT-1, the MSM was supplemented with DDT as the sole carbon and energy source. Each flask was inoculated with a suspension of the isolate DDT-1 at a biomass level of OD_600_ = 0.2. All flasks were incubated in a shaker at 150 rpm in the dark. The whole culture was sampled for the determination of DDT residues immediately after inoculation and after 3, 7, 14, and 21 days. Each treatment was performed in triplicate, and the control experiment without the isolate DDT-1 was performed under the same conditions. To determine the effect of DDT concentrations on its biodegradation, the medium (pH 7.0) was fortified with DDT at three levels of 0.1, 1.0, and 10.0 mg/l, respectively. To measure the effect of pH on the biodegradation, the medium was prepared with buffers at pH 5.0, 7.0, and 9.0, respectively (see Supplementary Information for components). To confirm the effect of temperature on the biodegradation, the medium (pH 7.0) was incubated at 15 °C, 25 °C, and 35 °C, respectively.

### Extraction and Determination of DDT and its metabolites

To reveal the degradation pathway of DDT by the isolate DDT-1, the experiment was carried out in a 100-ml Erlenmeyer flask containing 20 ml of MSM (pH 7.0) supplemented with 10.0 mg/l of DDT at an inoculation level of OD_600_ = 0.4. The extraction methods of DDT and its metabolites in MSM were included in Supplementary Information. DDT metabolites were identified using a GC-MS-QP2010 Plus equipped with electron impact (EI) ionization (Shimadzu Corporation, Kyoto, Japan) and a VF-1701MS silica capillary column (30 m × 0.25 mm × 0.25 μm, Agilent Technologies, USA)^13^.

### Recovery study

To confirm the validity of the extraction method of DDT, recovery studies were carried out at three spiking levels of 0.1, 1.0, and 10.0 mg/l in MSM. The recovery of DDT ranged from 92.2% to 101.5% with relative standard deviation ≤ 6.9% in MSM. The limit of detection (LOD) and limit of quantitation (LOQ) of DDT were 0.1 and 0.5 μg/l, respectively. These data indicate that the extraction method is satisfactory for the analysis of DDT residues.

### Nucleotide sequence deposite

The isolate DDT-1 has been submitted to CCTCC under M 2015153. The 16S rDNA sequence for the isolate DDT-1 has been deposited in the NCBI GenBank database under the accession number KP729429. The whole genome shotgun project has been deposited at DDBJ/EMBL/GenBank under the accession number LEKR00000000 (submission ID SUB972085, BioProject ID PRJNA286061, and BioSample ID SAMN03764479).

## Additional Information

**How to cite this article**: Pan, X. *et al.* Biodegradation of DDT by *Stenotrophomonas* sp. DDT-1: Characterization and genome functional analysis. *Sci. Rep.* **6**, 21332; doi: 10.1038/srep21332 (2016).

## Supplementary Material

Supplementary Information

## Figures and Tables

**Table 1:** Genome properties of the isolate DDT-1.

Property	Value	Property	Value
Raw reads size (bp)	1,767,899,136	sRNA total length (bp)	1795
Clean reads size (bp)	1,496,317,952	ncRNAs	1
Genome size (bp)	4,514,569	LTR number	27
Fold coverage	308×	LTR average length (bp)	79
GC content (%)	66.92	LTR total length (bp)	2140
Contigs (>500 bp)	97	LINE number	7
N50 length (bp)	80,532	LINE average length (bp)	137
N90 length (bp)	29,764	LINE total length (bp)	958
Max length (bp)	236,154	SINE number	14
Min length (bp)	510	SINE average length (bp)	64
Genes	4,108	SINE total length (bp)	892
Protein-coding genes	4,033	TRF number	221
Pseudo genes	75	TRF repeat size (bp)	3–621
Frameshifted genes	24	TRF total length (bp)	13,342
Protein-coding genes assigned to COGs	2,807	Minisatellite DNA number	188
KEGG mapping number	1,601	Minisatellite DNA repeat size (bp)	12–51
Gene average length (bp)	948	Minisatellite DNA total length (bp)	9485
Kmer depth	29	Microsatellite DNA number	10
tRNAs	67	Microsatellite DNA repeat size (bp)	3–6
tRNA average length (bp)	78	Microsatellite DNA total length (bp)	428
tRNA total length (bp)	5,160	5S rRNAs	3
sRNAs	14	16S rRNAs	4
sRNA average length (bp)	128	23S rRNAs	1

**Table 2:** Degradation kinetic data of DDT by the isolate DDT-1 at different substrate concentrations, temperatures, and pH values.

Concentration (mg/l)	Temperature (°C)	pH	Kinetic function	Degradation rate (mg/l/d)	DT_50_^*^ (d)	*r*^2^
0.1	30	7.0	C = 0.0937e^−0.1744*t^	0.004	4.0 a ^§^	0.84
1.0	30	7.0	C = 0.7941e^−0.0856*t^	0.038	8.1 b	0.92
10.0	30	7.0	C = 9.5835e^−0.0084*t^	0.086	82.5 c	0.92
1.0	15	7.0	C = 0.9098e^−0.03916*t^	0.022	17.7 d	0.97
1.0	25	7.0	C = 0.8911e^−0.04433*t^	0.024	15.6 e	0.98
1.0	35	7.0	C = 0.9322e^−0.06017*t^	0.034	11.5 f	0.99
1.0	30	5.0	C = 0.8839e^−0.05761*t^	0.032	12.0 f	0.97
1.0	30	7.0	C = 0.8707e^−0.0908*t^	0.038	7.6 b	0.91
1.0	30	9.0	C = 0.8761e^−0.06264*t^	0.033	11.1 f	0.97

^*^DT50: the degradation half-lives of DDT; ^§^: data followed by a different letter in the same column are significantly different (*p* ≤ 0.05).
