# Supplementary material for: Biodegradation of DDT by Stenotrophomonas sp. DDT-1: Characterization and genome functional analysis
Source: Sci Rep. 2016 Feb 18;6:21332. doi: 10.1038/srep21332 (PMC4758049; doi:10.1038/srep21332)
Supplement: Supplementary Information [file srep21332-s1.pdf]

Biodegradation of DDT by *Stenotrophomonas* sp. DDT-1: Characterization and genome functional analysis

Xiong Pan[1], Dunli Lin[1], Yuan Zheng[1], Qian Zhang[1], Yuanming Yin[1], Lin Cai[2], Hua Fang[1,*] & Yunlong Yu[1,*]

Extraction and determination of DDT metabolites. After incubation for 14 d under conditions of 30 ℃ and 150 rpm, the metabolites DDD, DDE, DDMS, and DDOH in MSM were extracted according to the method described by Fang et al.[1], which was the same extraction method with parent compound DDT. For the extraction of the metabolite DDA, the culture was firstly adjusted to pH 1-2 by 1 mol/l HCl, and then was extracted three times with 50, 40, and 30 ml ethyl acetate, respectively. The organic phase was dried through anhydrous sodium sulfate and collected in a 250-mL flat-bottom flask. It was subsequently concentrated to about 1 ml on a vacuum rotary evaporator and immediately concentrated to almost dryness under a gentle nitrogen flow. *n*-Hexane was added to dissolve DDA and the volume was made up to 10 ml and was filtered through a 0.22 μm syringe filter membrane. DDT metabolites were determined using a GC-MS-QP2010 Plus equipped with electron impact (EI) ionization (Shimadzu Corporation, Kyoto, Japan) and a VF-1701MS silica capillary column (30 m × 0.25 mm × 0.25 μm, Agilent Technologies, USA)[1]. The extraction and determination methods of DDT metabolites DDD, DDE, DDMS, DDOH, and DDA have been added in revised manuscript (please see lines 317-323 and Supplementary Information).

Reference:

1. Fang, H., Dong, B., Yan, H., Tang, F. F. & Yu, Y. L. Characterization of a bacterial strain

capable of degrading DDT congeners and its use in bioremediation of contaminated soil. *J. Hazard. Mater.* **184,** 281-289 (2010).

Buffer preparation. The medium was prepared with pH 5.0 buffer (KHP, 10.21 g; NaOH, 0.09 g; H_2O, 1000 ml), pH 7.0 buffer (MSM), and pH 9.0 buffer (KCl, 3.73 g; H_3BO_3, 0.93 g; NaOH, 0.83 g; H_2O, 1000 ml), respectively.

Statistical analysis. Mean and standard deviations of all data were processed using Microsoft Excel 2010 (Microsoft, Redmond, Washington, USA). The difference of DDT degradation by the isolate DDT-1 in MSM at different substrate concentration, pH, and temperature were analyzed by one-way ANOVA using SPSS version 15.0 (SPSS Inc., Chicago, Illinois, USA).

Inoculum preparation. The isolate DDT-1 was cultured in 250 ml Erlenmeyer flasks containing 150 ml LB medium (beef extract, 10 g; peptone, 5 g; sodium chloride, 5 g; distilled water, 1000 ml; pH 7.0) supplemented with DDT of 1 mg/l at 30 ℃ and 150 rpm on a rotary shaker.. At the exponential phase, the microorganisms were harvested by centrifugation (8000×g, 10 min). The bacterial sediment was washed three times with 20 ml of NaH2PO4-Na2HPO4 buffer (0.1 mol/l, pH 7.0), and then suspended in the same buffer and used to inoculate the degradation cultures.

Figure captions

Fig. S1 Growth curve of the strain DDT-1 in mineral salts medium (pH 7.0) supplemented with 1.0 mg/l of DDT at 30 ℃.

Tables

Table S1 16S rDNA sequence of the isolate DDT-1

Table S2 Functional annotation of strain DDT-6 genome against the Clusters of Orthologous Groups (COG) database

Table S3 Functional classification of strain DDT-6 genome against Gene Ontology (GO) database

Table S4 Metabolic annotation of strain DDT-6 genome against Kyoto Encyclopedia of Genes and Genomes (KEGG) pathway database

Table S5 Information on the assembled contigs from clean reads by SOAPdenovo

Table S6 DDGs-like genes found in strain DDT-1 genome using BLASTx

Table S7 Molecular weight and characteristic peak of DDT and its metabolites

Table S8 Composition of DDT degradation genes (DDGs) database

Fig. S1

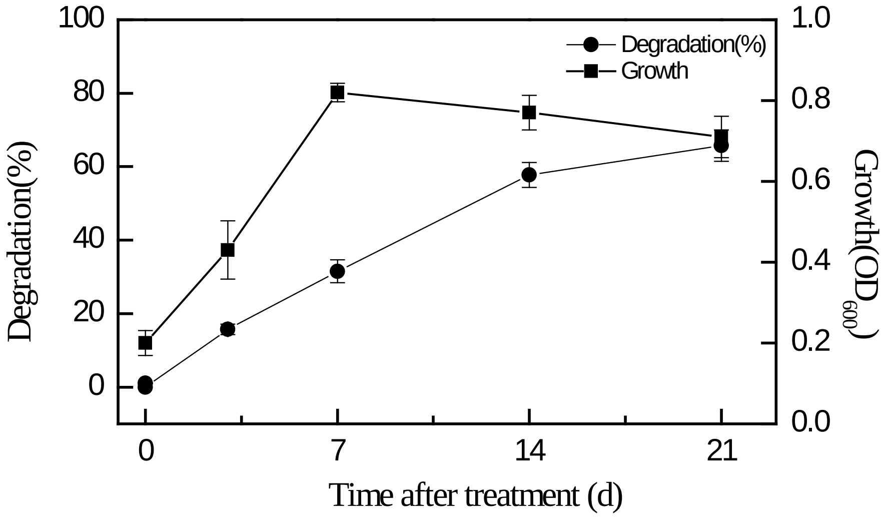

Degradation(%)
Growth
Degradation(%)
Growth(OD600)
Time after treatment (d)
0
7
14
21
0
20
40
60
80
100
0.0
0.2
0.4
0.6
0.8
1.0

Table S1 16S rDNA sequence of the isolate DDT-1

16S rDNA size (bp)	1422
Similarity	99%
Accession number	KP729429
Putive genus	*Stenotrophomonas*
16S rDNA sequence (bp)	GGCAGCGCCCTCCCGAAGGTTAAGCTACCTGCTTCTGGTGCAACAAACTCCCATGGTG<br>TGACGGGCGGTGTGTACAAGGCCCGGGAACGTATTCACCGCAGCAATGCTGATCTGC<br>GATTACTAGCGATTCCGACTTCATGGAGTCGAGTTGCAGACTCCAATCCGGACTGAG<br>ATAGGGTTTCTGGGATTGGCTTACCGTCGCCGGCTTGCAGCCCTCTGTCCCTACCATT<br>GTAGTACGTGTGTAGCCCTGGCCGTAAGGGCCATGATGACTTGACGTCATCCCCACCT<br>TCCTCCGGTTTGTCACCGGCGGTCTCCTTAGAGTTCCCACCATTACGTGCTGGCAACT<br>AAGGACAAGGGTTGCGCTCGTTGCGGGACTTAACCCAACATCTCACGACACGAGCTG<br>ACGACAGCCATGCAGCACCTGTGTTCGAGTTCCCGAAGGCACCCATCCATCTCTGGA<br>AAGTTCTCGACATGTCAAGGCCAGGTAAGGTTCTTCGCGTTGCATCGAATTAAACCAC<br>ATACTCCACCGCTTGTGCGGGCCCCCGTCAATTCCTTTGAGTTTCAGTCTTGCGACCGT<br>ACTCCCCAGGCGGCGAACTTAACGCGTTAGCTTCGATACTGCGTGCCAAATTGCACCC<br>AACATCCAGTTCGCATCGTTTAGGGCGTGGACTACCAGGGTATCTAATCCTGTTTGCT<br>CCCCACGCTTTCGTGCCTCAGTGTCAATGTTGGTCCAGGTAGCTGCCTTCGCCATGGA<br>TGTTCCTCCTGATCTCTACGCATTTCACTGCTACACCAGGAATTCCGCTACCCTCTACC<br>ACATTCTAGTCGTCCAGTATCCACTGCAGTTCCCAGGTTGAGCCCAGGGCTTTCACAA<br>CGGACTTAAATGACCACCTACGCACGCTTTACGCCCAGTAATTCCGAGTAACGCTTGC<br>ACCCTTCGTATTACCGCGGCTGCTGGCACGAAGTTAGCCGGTGCTTATTCTTTGGGTA<br>CCGTCATCCCAACCAGGTATTAGCCGGCTGGATTTCTTTCCCAACAAAAGGGCTTTAC<br>AACCCGAAGGCCTTCTTCACCCACGCGGTATGGCTGGATCAGGCTTGCGCCCATTGTC<br>CAATATTCCCCACTGCTGCCTCCCGTAGGAGTCTGGACCGTGTCTCAGTTCCAGTGTG<br>GCTGATCATCCTCTCAGACCAGCTACGGATCGTCGCCTTGGTGGGCCTTTACCCCGCC<br>AACTAGCTAATCCGACATCGGCTCATTCAATCGCGCAAGGCCCGAAGATCCCCTGCTT<br>TCACCCGTAGGTCGTATGCGGTATTAGCGTAAGTTTCCCTACGTTATCCCCCACGACA<br>GAGTAGATTCCGATGTATTCCTCACCCGTCCGCCACTCGCCACCCAGAGAGCAAGCTC<br>TCCTGTGCTGCCGTTCGACttGCATGtGttAG

Table S2 Functional annotation of strain DDT-6 genome against the Clusters of Orthologous Groups (COG) database

No.	Class description	Gene number
A	RNA processing and modification	1
B	Chromatin structure and dynamics	1
C	Energy production and conversion	178
D	Cell cycle control, cell division, chromosome partitioning	28
E	Amino acid transport and metabolism	218
F	Nucleotide transport and metabolism	56
G	Carbohydrate transport and metabolism	131
H	Coenzyme transport and metabolism	104
I	Lipid transport and metabolism	107
J	Translation, ribosomal structure and biogenesis	167
K	Transcription	203
L	Replication, recombination and repair	111
M	Cell wall/membrane/envelope biogenesis	158
N	Cell motility	89
O	Posttranslational modification, protein turnover, chaperones	124
P	Inorganic ion transport and metabolism	168
Q	Secondary metabolites biosynthesis, transport and catabolism	72
R	General function prediction only	360
S	Function unknown	242
T	Signal transduction mechanisms	147
U	Intracellular trafficking, secretion, and vesicular transport	100
V	Defense mechanisms	42

Table S3 Functional classification of strain DDT-6 genome against Gene Ontology (GO) database

Ontology	Class	Number
biological process	biological adhesion	10
Biological process	biological regulation	428
Biological process	cellular component organization or biogenesis	113
Biological process	cellular process	1513
Biological process	death	2
Biological process	developmental process	26
Biological process	establishment of localization	410
Biological process	localization	435
Biological process	locomotion	43
Biological process	metabolic process	1560
Biological process	multi-organism process	14
Biological process	multicellular organismal process	2
Biological process	negative regulation of biological process	9
Biological process	nitrogen utilization	2
Biological process	positive regulation of biological process	4
Biological process	regulation of biological process	419
Biological process	reproduction	9
Biological process	reproductive process	4
Biological process	response to stimulus	292
Biological process	signaling	175
Biological process	viral reproduction	4
Cellular component	cell	1448
Cellular component	cell part	1447
Cellular component	macromolecular complex	126
Cellular component	organelle	117
Cellular component	organelle part	35
Cellular component	virion	3
Cellular component	virion part	3
Molecular function	antioxidant activity	28
Molecular_function	binding	1178
Molecular function	catalytic activity	1582
Molecular function	enzyme regulator activity	4
Molecular function	molecular transducer activity	214
Molecular function	nucleic acid binding transcription factor activity	153
Molecular function	protein binding transcription factor activity	21
Molecular function	structural molecule activity	52
Molecular function	transporter activity	206

Table S4 Metabolic annotation of strain DDT-6 genome against Kyoto Encyclopedia of Genes and Genomes (KEGG) pathway database

No.	KEGG description	Number
map00010	Glycolysis/Gluconeogenesis	30
map00020	Citrate cycle (TCA cycle)	22
map00030	Pentose phosphate pathway	20
map00040	Pentose and glucuronate interconversions	9
map00051	Fructose and mannose metabolism	19
map00052	Galactose metabolism	10
map00053	Ascorbate and aldarate metabolism	4
map00061	Fatty acid biosynthesis	17
map00071	Fatty acid metabolism	14
map00072	Synthesis and degradation of ketone bodies	6
map00100	Steroid biosynthesis	1
map00120	Primary bile acid biosynthesis	1
map00121	Secondary bile acid biosynthesis	1
map00130	Ubiquinone and other terpenoid-quinone biosynthesis	9
map00190	Oxidative phosphorylation	47
map00195	Photosynthesis	8
map00230	Purine metabolism	61
map00240	Pyrimidine metabolism	45
map00250	Alanine, aspartate and glutamate metabolism	23
map00253	Tetracycline biosynthesis	3
map00260	Glycine, serine and threonine metabolism	26
map00270	Cysteine and methionine metabolism	28
map00280	Valine, leucine and isoleucine degradation	23
map00281	Geraniol degradation	8
map00290	Valine, leucine and isoleucine biosynthesis	20
map00300	Lysine biosynthesis	14
map00310	Lysine degradation	14
map00311	Penicillin and cephalosporin biosynthesis	5
map00312	beta-Lactam resistance	4
map00330	Arginine and proline metabolism	34
map00340	Histidine metabolism	21
map00350	Tyrosine metabolism	20
map00360	Phenylalanine metabolism	9
map00361	Chlorocyclohexane and chlorobenzene degradation	5
map00362	Benzoate degradation	11
map00363	Bisphenol degradation	8
map00364	Fluorobenzoate degradation	2
map00380	Tryptophan metabolism	17
map00400	Phenylalanine, tyrosine and tryptophan biosynthesis	23

map00401	Novobiocin biosynthesis	4
map00410	beta-Alanine metabolism	7
map00430	Taurine and hypotaurine metabolism	2
map00440	Phosphonate and phosphinate metabolism	4
map00450	Selenocompound metabolism	11
map00460	Cyanoamino acid metabolism	9
map00471	D-Glutamine and D-glutamate metabolism	2
map00473	D-Alanine metabolism	4
map00480	Glutathione metabolism	25
map00500	Starch and sucrose metabolism	21
map00510	N-Glycan biosynthesis	2
map00511	Other glycan degradation	2
map00520	Amino sugar and nucleotide sugar metabolism	31
map00521	Streptomycin biosynthesis	9
map00523	Polyketide sugar unit biosynthesis	4
map00524	Butirosin and neomycin biosynthesis	3
map00540	Lipopolysaccharide biosynthesis	12
map00550	Peptidoglycan biosynthesis	17
map00561	Glycerolipid metabolism	9
map00562	Inositol phosphate metabolism	5
map00564	Glycerophospholipid metabolism	22
map00565	Ether lipid metabolism	1
map00590	Arachidonic acid metabolism	5
map00591	Linoleic acid metabolism	6
map00592	alpha-Linolenic acid metabolism	2
map00620	Pyruvate metabolism	39
map00621	Dioxin degradation	1
map00623	Toluene degradation	5
map00624	Polycyclic aromatic hydrocarbon degradation	7
map00625	Chloroalkane and chloroalkene degradation	12
map00626	Naphthalene degradation	13
map00627	Aminobenzoate degradation	16
map00630	Glyoxylate and dicarboxylate metabolism	25
map00633	Nitrotoluene degradation	2
map00640	Propanoate metabolism	19
map00642	Ethylbenzene degradation	5
map00643	Styrene degradation	3
map00650	Butanoate metabolism	28
map00660	C5-Branched dibasic acid metabolism	7
map00670	One carbon pool by folate	16
map00680	Methane metabolism	26
map00710	Carbon fixation in photosynthetic organisms	11
map00720	Carbon fixation pathways in prokaryotes	25

map00730	Thiamine metabolism	5
map00740	Riboflavin metabolism	10
map00750	Vitamin B6 metabolism	7
map00760	Nicotinate and nicotinamide metabolism	16
map00770	Pantothenate and CoA biosynthesis	15
map00780	Biotin metabolism	9
map00785	Lipoic acid metabolism	2
map00790	Folate biosynthesis	13
map00791	Atrazine degradation	1
map00830	Retinol metabolism	3
map00860	Porphyrin and chlorophyll metabolism	15
map00900	Terpenoid backbone biosynthesis	12
map00903	Limonene and pinene degradation	12
map00908	Zeatin biosynthesis	1
map00910	Nitrogen metabolism	17
map00920	Sulfur metabolism	8
map00930	Caprolactam degradation	3
map00940	Phenylpropanoid biosynthesis	5
map00950	Isoquinoline alkaloid biosynthesis	1
map00960	Tropane, piperidine and pyridine alkaloid biosynthesis	4
map00970	Aminoacyl-tRNA biosynthesis	25
map00980	Metabolism of xenobiotics by cytochrome P450	14
map00982	Drug metabolism - cytochrome P450	14
map00983	Drug metabolism - other enzymes	8
map01040	Biosynthesis of unsaturated fatty acids	13
map01051	Biosynthesis of ansamycins	1
map01053	Biosynthesis of siderophore group nonribosomal peptides	7
map01055	Biosynthesis of vancomycin group antibiotics	1
map02010	ABC transporters	11
map02020	Two-component system	42
map02030	Bacterial chemotaxis	7
map02040	Flagellar assembly	1
map02060	Phosphotransferase system (PTS)	5
map03008	Ribosome biogenesis in eukaryotes	2
map03013	RNA transport	1
map03018	RNA degradation	12
map03020	RNA polymerase	4
map03030	DNA replication	14
map03060	Protein export	3
map03320	PPAR signaling pathway	4
map03410	Base excision repair	16
map03420	Nucleotide excision repair	7
map03430	Mismatch repair	17

map03440	Homologous recombination	15
map04011	MAPK signaling pathway - yeast	6
map04070	Phosphatidylinositol signaling system	5
map04112	Cell cycle - Caulobacter	6
map04113	Meiosis - yeast	1
map04122	Sulfur relay system	4
map04142	Lysosome	2
map04146	Peroxisome	10
map04260	Cardiac muscle contraction	3
map04614	Renin-angiotensin system	1
map04626	Plant-pathogen interaction	1
map04724	Glutamatergic synapse	2
map04910	Insulin signaling pathway	1
map04920	Adipocytokine signaling pathway	1
map04930	Type II diabetes mellitus	1
map04974	Protein digestion and absorption	1
map05010	Alzheimer's disease	5
map05012	Parkinson's disease	3
map05014	Amyotrophic lateral sclerosis (ALS)	3
map05016	Huntington's disease	8
map05020	Prion diseases	2
map05133	Pertussis	2
map05142	Chagas disease (American trypanosomiasis)	2
map05143	African trypanosomiasis	1
map05146	Amoebiasis	1
map05150	Staphylococcus aureus infection	1
map05152	Tuberculosis	1
map05211	Renal cell carcinoma	1
map05340	Primary immunodeficiency	1
map05410	Hypertrophic cardiomyopathy (HCM)	1

Table S5 Information on the assembled contigs from clean dataset by SOAPdenovo

Scaffold No.	Number	Length (bp)	GC content
Scaffold1	1	236154	66.47%
Scaffold2	1	204983	66.66%
Scaffold3	1	180975	67.34%
Scaffold4	1	173132	64.57%
Scaffold5	1	161570	67.53%
Scaffold6	1	151904	66.76%
Scaffold7	1	139431	65.93%
Scaffold8	1	128156	66.68%
Scaffold9	1	127164	66.51%
Scaffold10	1	125000	67.14%
Scaffold11	1	114231	62.78%
Scaffold12	1	111642	68.05%
Scaffold13	1	105178	66.67%
Scaffold14	1	94470	65.40%
Scaffold15	1	87475	67.07%
Scaffold16	1	84278	66.81%
Scaffold17	1	80532	66.77%
Scaffold18	1	79442	67.64%
Scaffold19	1	78162	68.15%
Scaffold20	1	76871	61.09%
Scaffold21	1	67002	67.38%
Scaffold22	1	65275	67.08%
Scaffold23	1	64187	67.24%
Scaffold24	1	62687	67.16%
Scaffold25	1	57491	67.01%
Scaffold26	1	54888	64.09%
Scaffold27	1	54550	67.32%
Scaffold28	1	54394	67.26%
Scaffold29	1	53271	67.01%
Scaffold30	1	53266	65.57%
Scaffold31	1	53165	68.17%
Scaffold32	1	51986	66.20%
Scaffold33	1	51608	66.38%
Scaffold34	1	50290	66.93%
Scaffold35	1	49599	66.36%
Scaffold36	1	48191	65.98%
Scaffold37	1	46771	67.43%
Scaffold38	1	46636	66.30%
Scaffold39	1	44950	67.41%
Scaffold40	1	43301	64.62%
Scaffold41	1	39070	67.53%

Scaffold42	1	37313	67.41%
Scaffold43	1	37308	66.94%
Scaffold44	1	35123	66.43%
Scaffold45	1	34954	67.69%
Scaffold46	1	34107	66.89%
Scaffold47	1	33543	66.60%
Scaffold48	1	33370	67.19%
Scaffold49	1	33154	66.05%
Scaffold50	1	33122	66.35%
Scaffold51	1	31936	66.99%
Scaffold52	1	31246	67.09%
Scaffold53	1	29834	67.11%
Scaffold54	1	29764	67.61%
Scaffold55	1	29309	67.90%
Scaffold56	1	27979	64.39%
Scaffold57	1	23952	67.20%
Scaffold58	1	22718	65.19%
Scaffold59	1	20945	66.72%
Scaffold60	1	18807	68.09%
Scaffold61	1	17418	68.13%
Scaffold62	1	17125	68.99%
Scaffold63	1	16941	59.47%
Scaffold64	1	16547	63.49%
Scaffold65	1	16088	67.88%
Scaffold66	1	15574	66.70%
Scaffold67	1	14109	68.17%
Scaffold68	1	13246	66.23%
Scaffold69	1	13194	65.36%
Scaffold70	1	13172	61.49%
Scaffold71	1	11817	68.82%
Scaffold72	1	11517	59.89%
Scaffold73	1	11426	68.93%
Scaffold74	1	11033	68.34%
Scaffold75	1	10210	67.77%
Scaffold76	1	9248	67.58%
Scaffold77	1	8799	69.20%
Scaffold78	1	8790	68.78%
Scaffold79	1	8168	67.64%
Scaffold80	1	7537	57.77%
Scaffold81	1	5712	66.84%
Scaffold82	1	5692	66.69%
Scaffold83	1	4799	68.26%
Scaffold84	1	2430	53.05%

Scaffold85	1	2264	54.24%
Scaffold86	1	1773	57.08%
Scaffold87	1	1522	58.41%
Scaffold88	1	1170	64.62%
Scaffold89	1	768	55.08%
Scaffold90	1	697	54.95%
Scaffold91	1	685	60.44%
Scaffold92	1	603	67.33%
Scaffold93	1	580	61.03%
Scaffold94	1	555	53.15%
Scaffold95	1	523	60.61%
Scaffold96	1	515	56.12%
Scaffold97	1	510	61.37%

Table S6 DDGs-like genes found in strain DDT-1 genome using BLASTx

**Query ID**	**Scaffold 1**	**Scaffold 2**	**Scaffold 3**	**Scaffold 9**	**Scaffold 10**	**Scaffold 17**	**Scaffold 18**	**Scaffold 31**	**Scaffold 52**
Subject id	CBW76073.1	CBW75398.1	CAQ47690.1	CAQ43870.1	CBW76470.1	CBW76142.1	CAQ43814.1	CAQ43710.1	CBW76219.1
Identity (%)	90.52	95.20	99.62	88.87	81.00	92.75	98.31	97.72	97.32
Alignment length	462	504	264	265	579	218	296	219	328
Mismatches	3	3	1	3	11	9	5	5	4
Gap openings	0	0	0	0	0	0	0	0	0
Query start	122407	27132	166404	122710	40748	19490	9018	45845	11948
Query end	123780	25627	165613	121916	39012	20131	8131	45189	10965
Subject start	1	1	1	1	1	11	1	1	114
Subject end	460	504	264	265	571	222	296	219	441
E-value	2e-156	0	2e-148	9e-125	0	9e-51	9e-168	7e-119	2e-99
Bit score	551	696	524	445	932	198	587	424	359
Metabolite pathway	DDNU ↓ DDOH	DDNU ↓ DDOH	DDA ↓ DDM	DDT ↓ DDE	DDT ↓ DDD	OHCTT ↓ CDP	DDMU ↓ DDOH	DDA ↓ DDM	OHCTT ↓ CDP
DDGs gene	*hdt*	*hdt*	*dcl*	*dhc*	*rdh*	*hdl*	*sds*、*dhg*	*dcl*	*hdl*

DDD: 1,1-dichloro-2,2-bis(p-chlorophenyl)ethane; DDE: 2,2-bis(p-chlorophenyl)-1,1-dichlorethylene;

DDMU: 1-chloro-2,2-bis(4'-chlorophenyl)ethylene; DDOH: 2,2-bis(4'-chlorophenyl)ethanol;

DDNU: unsym-bis(4'-chlorophenyl)ethylene; CDP: 2-(4'-chlorophenyl)-3,3-dichloropropenoate;

DDA: bis(4'-chlorophenyl)acetate; DDM: bis(4'-chlorophenyl)methane;

OHCTT: 6-Oxo-2-hydroxy-7-(4'-chlorophenyl)-3,8,8-trichloroocta-2E,4E,7E-trienoate.

Table S7 Molecular weight and characteristic peak of DDT and its metabolites

**Compound**	**Molecular weight**	**Characteristic peak**
DDT	354.5	235, 165
DDD	320	235, 165
DDE	318	246, 176
DDMS	266	212, 176
DDOH	266	235, 165
DDA	282	235, 165

Table S8 Composition of DDT degradation genes (DDGs) database

No.	Gene	Degradation pathway	Enzyme	Protein number
1	*dhc*	DDT→DDE	dehydrochlorinase	10
2	*rrat*	DDE→DDMU	ribonucleotide reductase activating transmembrane protein	2
3	*sds*	DDMU→DDMS	reductase	25
4	*cpo*	dhdDDT→dhDDT	coproporphyrinogen III oxidase	45
5	*doa*	DDT→dhdDDT	2,3-dioxygenase	35
6	*dcl*	DDA→DDM	decarboxylase	253
7	*ort*	dhDDT→OHCTD	oxidoreductase	83
8	*ort*	DDCCE→OHCTT	oxidoreductase	83
9	*hdl*	OHCTT→CDP	hydrolase	168
10	*ods*	CBA→CB	oxidase	1
11	*rdh*	DDT→DDD	reductive dehalogenase	2
12	*dhc*	DDD→DDMU	dehydrochlorinase	10
13	*dhg*	DDMS→DDOH	dehalogenase	45
14	*dhc*	DDMS→DDNU	dehydrochlorinase	10
15	*hdt*	DDNU→DDOH	hydratase	210

DDT: 1,1,1-trichloro-2,2-bis(p-chlorophenyl)ethane;
DDD: 1,1-dichloro-2,2-bis(p-chlorophenyl)ethane;
DDE: 2,2-bis(p-chlorophenyl)-1,1-dichlorethylene;
DDMU: 1-chloro-2,2-bis(4'-chlorophenyl)ethylene;
DDMS: 1-chloro-2,2-bis(4'-chlorophenyl)ethane;
DDOH: 2,2-bis(4'-chlorophenyl)ethanol;
DDNU: unsym-bis(4'-chlorophenyl)ethylene;
CB: 4-chlorobenzoate;
CBA: 4-chlorobenzaldehyde;
CDP: 2-(4'-chlorophenyl)-3,3-dichloropropenoate;
DDA: bis(4'-chlorophenyl)acetate;
DDM: bis(4'-chlorophenyl)methane;
dhdDDT: cis-2,3-dihydrodiol-DDT;
dhDDT: 2,3-dihydroxy-DDT;
OHCTT: 6-Oxo-2-hydroxy-7-(4'-chlorophenyl)-3,8,8-trichloroocta-2E,4E,7E-trienoate;
OHCTD: 6-Oxo-2-hydroxy-7-(4’-chlorophenyl)-3,8,8,8-tetrachloroocta-2E,4E-dienoate;
DDCCE: 1,1-dichloro-2-(dihydroxy-4'-chlorophenyl)-2-(4'-chlorophenyl)ethylene.
